# Plasmapheresis Responsive Rapid Onset Dementia with Predominantly Frontal Dysfunction in the Context of Hashimoto’s Encephalopathy

**DOI:** 10.3389/fpsyt.2017.00212

**Published:** 2017-10-26

**Authors:** Dominique Endres, Magnus S. Vry, Petra Dykierek, Anne N. Riering, Eva Lüngen, Oliver Stich, Rick Dersch, Nils Venhoff, Daniel Erny, Irina Mader, Philipp T. Meyer, Ludger Tebartz van Elst

**Affiliations:** ^1^Section for Experimental Neuropsychiatry, Department of Psychiatry and Psychotherapy, Medical Center – University of Freiburg, Faculty of Medicine, University of Freiburg, Freiburg, Germany; ^2^Department of Neurology, Medical Center – University of Freiburg, Faculty of Medicine, University of Freiburg, Freiburg, Germany; ^3^Department of Rheumatology and Clinical Immunology, Medical Center – University of Freiburg, Faculty of Medicine, University of Freiburg, Freiburg, Germany; ^4^Institute of Neuropathology, Medical Center – University of Freiburg, Faculty of Medicine, University of Freiburg, Freiburg, Germany; ^5^Berta-Ottenstein-Programme, Medical Center – University of Freiburg, Faculty of Medicine, University of Freiburg, Freiburg, Germany; ^6^Department of Neuroradiology, Medical Center – University of Freiburg, Faculty of Medicine, University of Freiburg, Freiburg, Germany; ^7^Department of Nuclear Medicine, Medical Center – University of Freiburg, Faculty of Medicine, University of Freiburg, Freiburg, Germany

**Keywords:** Hashimoto’s encephalopathy, SREAT, frontotemporal dementia, plasmapheresis, thyroid

## Abstract

**Background:**

Hashimoto’s encephalopathy (HE) is a rare immunological neuropsychiatric disorder characterized by increased antithyroid antibodies and mixed neurological and psychiatric symptoms. HE has been previously discussed as a differential diagnosis for rapid progressive dementia. However, most of these patients suffered from additional neurological symptoms, like ataxia or seizures.

**Case presentation:**

Here, we present the case of a 59-year-old female patient suffering rapid onset dementia with salient frontal executive dysfunction. She developed rapid onset symptoms, including apathy, verbal depletion up to a stuporous state, severe working memory deficits, evidence of primitive reflexes, disturbed Luria’s three-step test, and micturition disorder. Analysis of her cerebrospinal fluid was normal. The serum analyses showed increased antithyroid (antithyroid peroxidase and antithyroglobulin) antibodies. In the cerebral magnetic resonance imaging, supratentorial deep and peripheral white matter lesions were found; the electroencephalography showed intermittent slowing, and the [^18^F]fluorodeoxyglucose positron emission tomography (FDG-PET) depicted medial and superior dorsolateral frontal hypometabolism. Several different psychopharmacological therapeutic approaches with various neuroleptics, antidepressants, and high doses of lorazepam were unsuccessful. Due to the organic alterations, including increased antithyroid antibodies, HE was suspected. Against expectations, treatment with high-dose corticosteroids proved to be ineffective and was associated with worsening symptoms. However, escalated treatment with plasmapheresis over 5 days led to significant improvement in all reported symptoms and in psychometric testing. The neuropsychological improvement was stable over a 6-month follow-up period, and the FDG-PET normalized.

**Conclusion:**

This case report reveals that (1) HE can mimic rapid onset dementia with predominantly frontal dysfunction; (2) this syndrome can be successfully treated in the context of HE; and (3) plasmapheresis can be effective in such a disease constellation. The detection of the immunological causes of rapid onset dementia and other psychiatric syndromes is important because it opens opportunities for new, innovative immunosuppressive treatment options.

## Background

Hashimoto’s encephalopathy (HE) is a rare immunological neuropsychiatric disorder that is typically characterized by mixed neurological (seizures in 47%, speech disorders in 37%, gait disturbances in 27%, myoclonic jerks in 22%, headaches in 16%) and psychiatric symptoms [confusion in 46%, memory disturbances in 43%, delusions in 25%, depression in 12% (1)]. In addition, isolated schizophreniform, depressive, or bipolar syndromes have been described at single-case levels (2–6). The link between HE and rapid progressive dementia has been previously described; however, most of these patients also suffered from seizures or other neurological symptoms (e.g., ataxia or gait disturbances). Therefore, HE was discussed as a differential diagnosis for Creutzfeldt–Jakob disease (7–9). In single cases, HE has also presented with isolated dementia (10).

Hashimoto’s encephalopathy should be considered if neuropsychiatric symptoms occur together with autoimmune thyroiditis, increased antithyroid [antithyroid peroxidase (anti-TPO) or antithyroglobulin (anti-TG)] antibodies, cerebrospinal fluid (CSF; mostly blood–brain barrier dysfunction), electroencephalography (EEG; mostly encephalopathic patterns), and cerebral magnetic resonance imaging (cMRI; mostly white matter lesions) alterations (1, 11, 12). Steroid treatment has been successful in most cases; therefore, in such constellations, HE is also called steroid-responsive encephalopathy associated with autoimmune thyroiditis (SREAT). Little information is available for second-line treatment alternatives, like plasmapheresis (1).

## Case Presentation

Here, we present the case of a 59-year-old woman who developed a rapid onset dementia with salient frontal executive dysfunction beginning in February 2016. One month earlier, she had shown prodromal symptoms with a decreased energy level, increased rumination, sleep disturbances, and a loss of appetite with weight loss. No psychoactive causes were identified. In February 2016, she showed rapid worsening of symptoms with increasing loss of interest in daily activities, withdrawal from other people, and reduced spontaneous speech output. Over the next few days, she developed a stuporous state with catatonic features; she lost all personal initiative and moved rarely, her movements appeared to be frozen, she stopped speaking, and she stared blankly. Since then, she has no longer been able to communicate adequately. Verbal exchanges were reduced to answers to questions using one or two words and dramatically prolonged response latencies. Pharmacological treatment with various antidepressants (citalopram up to 20 mg/day, venlafaxine up to 150 mg/day), neuroleptics (amisulpride up to 400 mg/day, aripiprazole up to 15 mg/day, flupentixol up to 1.5 mg/day, quetiapine up to 200 mg/day, risperidone up to 4 mg/day), anxiolytics (lorazepam up to 5 mg/day), and methylprednisolone (5 mg × 500 mg for five consecutive days for presumed SREAT) were administered over the course of more than 6 months, until September 2016. However, all these approaches were unsuccessful. Steroid treatment led to worsening apathy and cognitive slowing. Therefore, the patient was continuously treated in an inpatient setting.

Upon admission to our clinic (September 2016), she was still in a stuporous state and her perception, concentration, attention, and working memory were severely disturbed. She was aware of her state but negated emotional involvement. Mental fluency and judgment were compromised. She had no energy and was apathetic. However, hallucinations and delusional symptoms were not reported. The neurological examination showed evidence of primitive reflexes (orbicularis oris reflex) and a significantly disturbed Luria’s three-step test. In addition, the patient reported a new micturition disorder.

### Developmental, Somatic, and Family History

This patient’s developmental history was negative for *in utero* or birth complications, febrile convulsions, inflammatory brain diseases, and cerebral contusions. There was no evidence of any neurodevelopmental or personality disorders. The premorbid personality was described as vivacious, cheerful, and outgoing. She smoked but did not consume alcohol or illegal drugs. Until the onset of the symptoms at age 58, she was mentally healthy. Her somatic medical history included only complex regional pain syndrome of the right upper extremity (in 2005) and Hashimoto thyroiditis. Her family history of neuropsychiatric or malignant diseases was unremarkable.

### Investigations (Before Plasmapharesis)

**Figure 1:**
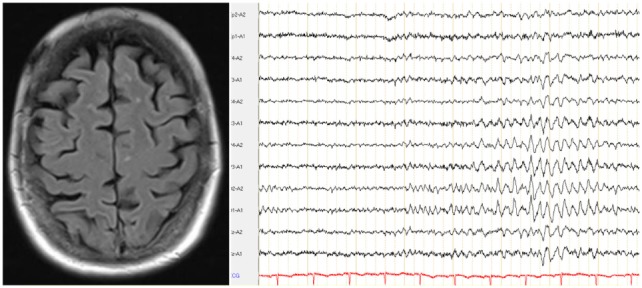
Diagnostic results. The cerebral magnetic resonance imaging demonstrated juxtacortical white matter lesions; the electroencephalography showed intermittent slowing.

**Figure 2:**
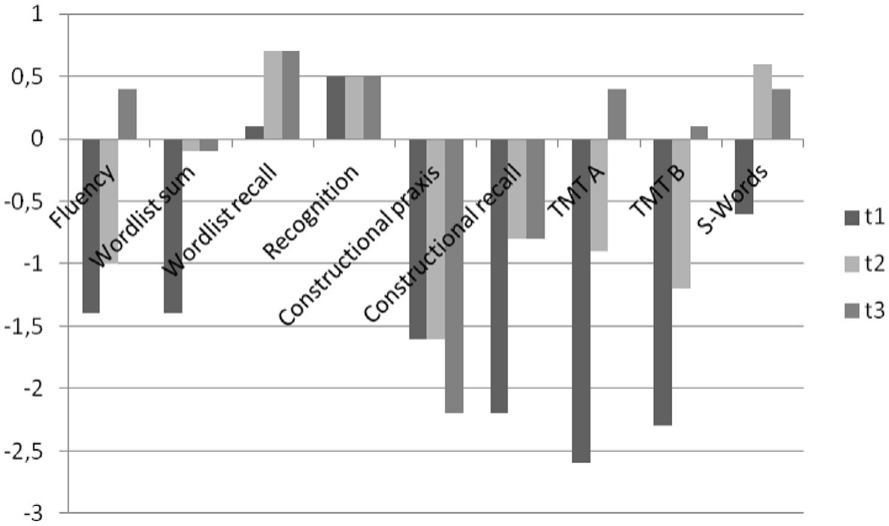
The course of the patient’s memory dysfunction using the CERAD (*z*-values). TMT A/B, trailmaking tests A/B; t1, before plasmapharesis; t2, 1 month after plasmapheresis; t3, nearly 6 months after plasmapheresis.

This patient’s serum anti-TPO and anti-TG antibodies were increased; however, no antineuronal antibodies against intracellular antigens were found. The CSF analyses showed normal findings; antibodies against neuronal cell surface antigens and markers of dementia were negative. A screening for rheumatological autoantibodies was negative. The cMRI showed multiple lesions in deep and peripheral white matter whithout diffusion restriction or contrast enhancement, while intermittent slow activity was detected in the EEG (Figure 1). [^18^F]fluorodeoxyglucose positron emission tomography (FDG-PET) showed mild-to-moderate medial and superior dorsolateral frontal hypometabolism, which did not allow a clear distinction between early-stage frontotemporal lobar degeneration and secondary non-specific changes (e.g., due to reduced consciousness/apathy, atrophy). An additional [^123^I]FP-CIT-SPECT revealed a normal striatal dopamine transporter availability (Table 1). Neuropsychological test batteries for dementia, following the Consortium to Establish a Registry for Alzheimer’s Disease (CERAD), showed deficits in verbal fluency, word list memory, constructional praxis, and trailmaking A/B (Figure 2). The Behavioral Assessment of the Dysexecutive Syndrome (BADS) showed severe impairment (sum-score: 6; average: 16–20; range: 0–24). More complex tasks, like the zoo map test and the modified six elements test, were nonexecutable, as the patient did not understand the instructions.

**Table 1:** Diagnostic findings.

Serum analyses	Thyroid-stimulating hormone (TSH) level was suppressed (0.06 mU/mL; reference 0.27–4.20 mU/mL); triiodothyronine (3.57 pmol/l; reference 3.4–6.8 pmol/l), and thyroxine (20.7 pmol/l; reference 10.6–22.7 pmol/l) levels were in normal ranges. Increased autoantibodies against thyroglobulin (832 IU/mL; reference < 115 IU/mL) and thyroid peroxidase (84 IU/mL; reference < 34 IU/mL) were detected. Autoantibodies against TSH receptors were not increased (0.93 IU/mL). No antibodies against intracellular onconeural antigens (Yo, Hu, CV2/CRMP5, Ri, Ma1/2, SOX1) or intracellular synaptic antigens (GAD, amphiphysin) were found. Screening for antinuclear antibodies (ANA), anti-neutrophil cytoplasmic antibodies (ANCA), antiphospholipid antibodies (APA), and rheumatoid factor (RF) was negative. C3, C4, and C3d were normal.
Cerebrospinal fluid analyses	Normal white cell count (2/μL; reference < 5/μL). No blood–brain barrier dysfunction (protein concentration: 401 mg/L; reference < 450 mg/L; albumin quotient: 5.3; age-dependent reference < 8 × 10^–3^). No CSF specific oligoclonal bands; IgG index not increased (0.44; reference ≤ 0.7). Antibodies against neuronal cell surface antigens (*NMDAR, AMPA-R, GABA-B-R, VGKC-complex [LGI1, Caspr2]*) were negative. Dementia markers were normal: Tau: 118 pg/mL (reference < 450 pg/mL), phospho-tau: 26 pg/mL (reference < 61 pg/mL), Aß 1–42: 1064 pg/mL (reference > 450 pg/mL, Aß ratio: 1.4 (reference > 0.5)
Cerebral magnetic resonance imaging	Supratentorial deep and peripheral white matter lesions (Fazekas Score 1). No generalized or local atrophy.
Electroencephalography	Intermittent rhythmic slow activity; no epileptic patterns.
Fluorodeoxyglucose positron emission tomography (FDG-PET)	Mild-to-moderate medial and superior dorsolateral frontal hypometabolism. Whole-body FDG-PET/CT for tumor screening was unremarkable.
FP-CIT single-photon emission computed tomography	Normal striatal dopamine transporter availability.

### Differential Diagnosis

The dysexecutive syndrome (e.g., loss of motivation and ability to judge), cognitive deficits (e.g., working memory deficits), neurological signs (orbicularis oris reflex, Luria’s three-step test), new urinary incontinence, neuropsychological testing, and frontal hypometabolism on FDG-PET were all compatible with a behavioral variant of frontotemporal dementia (FTD). However, the initial clinical course with rapid deterioration within days to weeks was highly unusual for neurodegenerative pathology like FTD; and the trailmaking tests A/B and verbal fluency (in the CERAD) would be adversely affected by the patient’s prevailing conscious state. Therefore, the patient’s conscious state might have influenced the comparability of the serial neuropsychological assessments. However, the cooperation was comparable at the three times of investigation. Alternatively, immunological encephalopathy seemed to be plausible regarding the rapid onset, increased antithyroid antibodies, EEG slowing, white matter lesions, and secondary non-specific FDG-PET alterations. Furthermore, an idiopathic depressive stupor could not be completely ruled out; however, the combination of slowed EEG and frontal FDG-PET hypometabolism is atypical for this differential diagnosis.

### Treatment Considerations

For optimal antidepressant treatment, lithium therapy was started in September 2016 (450–675 mg/d, serum levels: 0.39–0.69 mmol/l, reference: 0.4–0.8 mmol/l). There was only a questionable minor response to this treatment attempt over a period of 7 weeks. In parallel, we discussed options for immunosuppressive treatment strategies. Although high-dose steroid therapy aggravated symptoms, we started off-label treatment with plasmapheresis (five sessions at the beginning of November 2016) 7 weeks after the initiation of lithium as a last-resort therapeutic attempt. At this time point, the patient’s hypothyroidism was adequately substituted with 0.075 mg/d l-thyroxine.

### Outcome and Follow-Up

**Figure 3:**
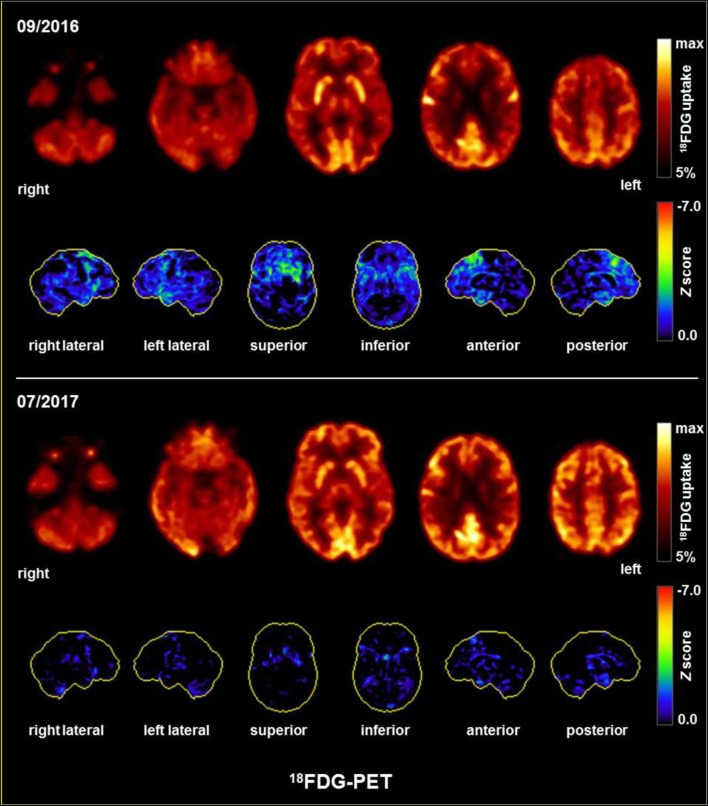
FDG-PET showing mild-to-moderate medial and superior dorsolateral frontal hypometabolism before and normalization after plasmapheresis. The upper and lower row images show the transaxial fluorodeoxyglucose positron emission tomography (FDG-PET) images and the 3D surface projections of the regions with decreased FDG uptake (color-coded *Z*-score and compared with age-matched healthy controls, respectively).

A few days after the initiation of plasmapheresis, all the patient’s symptoms improved significantly. Although her oral fluency was still reduced, normal communication was again possible. Her mood improved, her cognitive deficits were reduced, the orbicularis oris reflex disappeared, and the Luria’s three-step test normalized. However, her energy level improved only slightly. This patient was discharged from our clinic in December 2016 (6 weeks after undergoing plasmapheresis) and lived with her husband as before. Her energy levels and micturition disorder gradually improved; however, while her oral fluency and planning skills were enhanced, she still showed slight impairment 6 months after plasmapheresis. The improvement was neuropsychologically documented (Figure 2). One month after plasmapheresis, the patient improved in almost all the initially impaired categories of the CERAD testing battery (Figure 2); however, the BADS only improved slightly and remained significantly below average (sum score only increased from 6 to 8). Nearly 6 months after undergoing plasmapheresis, the CERAD (Figure 2) and BADS (sum score of 8 points) findings were still stable. However, the verbal fluency was slightly reduced. Treatment with lithium (675 mg/d) and l-thyroxine (0.075 mg/d) continued unchanged. The follow-up FDG-PET showed normalization in July 2017 (i.e., the frontal hypometabolism was no longer detectable; Figure 3).

## Discussion

In this paper, we present the case of a female patient with rapid onset encephalopathy in the context of HE successfully treated with plasmapheresis. This case report is important because it shows that dementia with salient frontal executive dysfunction can be successfully treated in the context of HE. Furthermore, the case report demonstrates that plasmapheresis could be effective in a similar disease constellation, even after high-dose steroid treatment proved to be ineffective.

### Diagnostic Considerations

Hashimoto’s encephalopathy was suspected in our patient due to the increased anti-TPO and anti-TG antibodies, rapid symptom onset, EEG slowing, white matter lesions in the cMRI, and (possibly only secondary) non-specific frontal hypometabolism on FDG-PET. The CSF, which shows a blood–brain barrier dysfunction in over 80% of HE patients (1), was normal in our patient. However, all the alterations found in our patient were unspecific. In previous studies, antithyroid antibodies have been found in 13% of healthy adults [and even more often in females and older individuals (13, 14)]; EEG slowing was found in different psychiatric disorders [and infrequently in healthy controls (15, 16)]; a small number of cerebral hemispheric white matter lesions is a common incidental finding in healthy adults of this age (17). Only the patient’s responsiveness to plasmapheresis suggested the immunological cause. The clinical improvement had an extremely close temporal relation with the plasmapheresis; therefore, we interpreted this as an effect of the plasma exchange. However, as a limiting aspect, lithium therapy was started 7 weeks prior to the plasma exchange; therefore, it cannot be ruled out that a late lithium effect in the context of a depressive stupor contributed to the improvement. In our opinion, the significant improvement in direct succession to plasma exchange, the severe clinical manifestation with temporal and frontal signs and symptoms (CERAD, perception disorder, orbicularis oris reflex, abnormal Luria’s three-step test, urine incontinence), and the absence of significant improvement following high-dose lorazepam treatment or 7 weeks of lithium treatment contradict a depressive stupor and a relevant lithium effect. The subacute course and the rapid clinical improvement under therapy are contradictory to FTD.

### Pathophysiological Considerations

Our recent findings of intrathecal antithyroid antibody synthesis in a subgroup of autoantibody positive patients, as well as the increased antibody indices in HE patients in a study from the Ferracci group, support the idea of central autoimmunity (18–20). However, it is still unclear whether these autoantibodies play a direct etiopathogenic role or whether they are an epiphenomenon of the autoimmune process, similar to the measles–rubella–varicella zoster (MRZ) virus reaction in patients with multiple sclerosis (21). Most authors have favored the idea of an epiphenomenon because the autoantibody titers did not correlate with the severity of clinical symptoms. The detection of cross-reactivity between the thyroid gland and brain epitopes could support the hypothesis of a direct etiopathogenic role of antithyroid autoantibodies. Blanchin et al. (22) demonstrated antibody binding (i.e., for anti-TPO antibodies) to cerebellar tissue, whereas Moodley et al. (23) showed anti-TG antibodies bound only to cerebral vessels. However, these findings have yet to be reproduced. Taken together, the exact role of antithyroid antibodies remains unclear, and this field requires further research. Alternatively, our patient’s symptoms might also be due to yet unknown or unidentified antineuronal antibodies. For a better pathophysiological understanding, cytokine profiling should also be performed in further patients with HE.

The positive effect of plasmapheresis (i.e., the procedure of separating the blood, exchanging the plasma, and returning the other components to the patient) in our patient might have been due to the removal of the circulating antithyroid antibodies or perhaps other yet unknown autoantibodies in HE patients or, alternatively, to the elimination of immune complexes, components of the complement cascade, cytokines, and/or other inflammatory mediators (24, 25). The successful application of plasmapheresis has also been described in a review of 10 cases with neurological forms of HE, in which nine patients (90%) showed clinical improvement (26). We discussed with our patient that plasmapheresis would be necessary again in the case of clinical deterioration. From an electrophysiological perspective, a reduction in the inflammatory brain processes related to the plasmapheresis might secondarily stabilize the cerebral networks and, therefore, reduce the clinical symptoms due to local area network inhibition [LANI hypothesis (27–29)].

### Clinical Importance of Such Case Studies

Our case report illustrates the importance of a thorough diagnostic workup in dementia, including cMRI, EEG, FDG-PET, CSF analysis, and autoantibody testing. The detection of HE and other immunological encephalopathies is highly relevant because of the potential therapeutic implications. Unfortunately, for the alternative diagnosis of idiopathic FTD, no treatment is available. In the presence of increased antithyroid antibodies, individual diagnostic considerations should include extensive CSF and instrument-based diagnostics. From a therapeutic perspective, therapy with corticosteroids is the established first-line intervention. However, if proven to be unsuccessful, alternative treatment options, like plasmapheresis or intravenous immunoglobulins, could be considered (30, 31). We have previously published two cases of HE mimicking schizophreniform and affective disorders, demonstrating the potential importance of this intervention in HE as a potentially treatable disorder mimicking classical psychiatric phenotypes (3, 6). As the next step in investigating this link, case series should be performed to detect the frequency and the characteristics of isolated psychiatric manifestations of HE.

## Conclusion

Single patients with rapid onset encephalopathy in the context of increased antithyroid antibodies may be treated successfully with plasmapheresis, even if first-line corticosteroids treatment proves to be unsuccessful or even unfavorable. We suggest that screening for antithyroid antibodies should become a routine procedure among patients with rapid onset dementia. However, the decision to administer immunosuppressive therapy in the presence of increased antithyroid antibodies should consider other organic alterations based on findings in a thorough neuropsychiatric workup, including CSF, EEG, cMRI, and FDG-PET studies. Overall, the detection of immunological forms of dementia and other psychiatric syndromes allows new and innovative immunosuppressive treatment options.

## Ethics Statement

The patient has given her signed written informed consent for this case report, including the presented images, to be published.

## Author Contributions

DEn, MV, and LTvE treated the patient. DEn and LTvE performed the data research. MV and EL supported the data research. DEn wrote the paper. IM performed the cMRI analyses. RD and OS performed the EEG and CSF analyses. NV performed the rheumatological analyses. PD and AR performed the neuropsychological testing. DEr and MV helped to draft the manuscript. All authors were critically involved in the theoretical discussion and composition of the manuscript. All authors read and approved the final version of the manuscript.

## Conflict of Interest Statement

DEn: None. MSV: None. PD: None. ANR: None. EL: None. OS: Consulting and lecture fees, grant and research support from Bayer Vital GmbH, Biogen Idec, Genzyme, Merck Serono, Novartis, Sanofi-Aventis and Teva. RD: None. NV: Advisory boards, lectures, research or travel grants within the last three years: Janssen-Cilag, Roche, Novartis, AbbVie, GSK, Medac, Pfizer. DEr: None. IM: Lecture fees from UCB Pharma GmbH, Germany. PTM: None. LTvE: Advisory boards, lectures, or travel grants within the last three years: Eli Lilly, Janssen-Cilag, Novartis, Shire, UCB, GSK, Servier, Janssen, and Cyberonics.
